# Poster Session I - A90 INVESTIGATING THE ROLE OF OREXIN SIGNALING IN IMPAIRED SATIETY SENSING IN OBESITY.

**DOI:** 10.1093/jcag/gwaf042.090

**Published:** 2026-02-13

**Authors:** O I DiPaolo, A Chakraborty, S Sachdev, D Reed

**Affiliations:** Queen’s University, Kingston, ON, Canada; Queen’s University, Kingston, ON, Canada; Queen’s University, Kingston, ON, Canada; Queen’s University, Kingston, ON, Canada

## Abstract

**Background:**

Obesity is linked to overeating which is driven by impaired satiety sensing. Satiety is conveyed from the gut to the brain by vagal afferent neurons (VANs) with cell bodies in the nodose ganglia (NG). In obesity, there is evidence of diminished satiety sensation mediated by decreased NG neuronal excitability.

**Aims:**

We aimed to determine how luminal mediators from obese mice contribute to these changes.

**Methods:**

Male and female C57BL/6 mice were fed a high-fat diet (HFD, 60% kCal from fat) or low-fat diet for 5 weeks (LFD, 6% kCal from fat) (HFD N = 6, LFD N = 7-8 per group). Fecal and jejunal contents from HFD and LFD mice were used to produce supernatants. Naïve NG neurons (N = 11-12 cells per group) were incubated with these supernatants and perforated patch clamp recordings were performed the next day to measure rheobase (higher rheobase = decreased excitability). Male and female HFD or LFD (N = 8 per group) fed mice received daily intraperitoneal (IP) injections of 10 mg/kg orexin-1 receptor (OX1R) antagonist (SB-334867) for one week to assess changes in food intake and body weight.

**Results:**

NG neurons from HFD mice had 73% higher rheobase than LFD mice (51.76 ± 14.68 pA vs. 71.33 ± 15.06 pA, p < 0.001). Naïve NG neurons incubated with HFD supernatants had 50% greater rheobase than LFD (100.0 ± 32.25 pA vs. 63.08 ± 15.48 pA, p < 0.05), an effect blocked by 10 µM OX1R antagonist (p < 0.01). HFD mice receiving OX1R antagonist had a 55% decrease in daily food intake compared to baseline (0.30 ± 0.05 vs. 0.45 ± 0.10 kCal/g body weight, p < 0.001) and an 8% difference in body weight gain (4.71% ± 0.91% vs. -3.09% ± 0.70%, p < 0.0001). LFD mice showed no significant changes in food intake or body weight in response to the antagonist.

**Conclusions:**

Supernatants from HFD mice decreased NG neuron excitability, which can be prevented by an OX1R antagonist. Administration of OX1R antagonist *in vivo* reduces food intake and weight gain in HFD but not LFD mice, suggesting orexin receptors play a role in obesity-related satiety impairment.

**Funding Agencies:**

None

